# Structural Characterization and Properties of Modified Soybean Meal Protein via Solid-State Fermentation by *Bacillus subtilis*

**DOI:** 10.3390/molecules28248015

**Published:** 2023-12-08

**Authors:** Xinyu Miao, Honghong Niu, Mubai Sun, Da Li, Mei Hua, Jinghui Wang, Ying Su

**Affiliations:** Institute of Agro-Food Technology, Jilin Academy of Agricultural Sciences (Northeast Agricultural Research Center of China), Changchun 133000, China; miaoxinyu@cjaas.com (X.M.); nhh29852@cjaas.com (H.N.); sunmubai@cjaas.com (M.S.); jacken0000@163.com (D.L.); huamei@cjaas.com (M.H.)

**Keywords:** solid-state fermentation, soybean meal, *Bacillus subtilis*, protein structure, protein properties

## Abstract

Soybean meal (SBM) is a high-quality vegetable protein, whose application is greatly limited due to its high molecular weight and anti-nutritional properties. The aim of this study was to modify the protein of soybean meal via solid-state fermentation of *Bacillus subtilis*. The fermentation conditions were optimized as, finally, the best process parameters were obtained, namely fermentation temperature of 37 °C, inoculum amount of 12%, time of 47 h, and material-liquid ratio of 1:0.58, which improved the content of acid-soluble protein. To explore the utilization of modified SBM as a food ingredient, the protein structure and properties were investigated. Compared to SBM, the protein secondary structure of fermented soybean meal (FSBM) from the optimal process decreased by 8.3% for α-helix content, increased by 3.08% for β-sheet, increased by 2.71% for β-turn, and increased by 2.51% for random coil. SDS-PAGE patterns showed that its 25–250 KDa bands appeared to be significantly attenuated, with multiple newborn peptide bands smaller than 25 KDa. The analysis of particle size and zeta potential showed that fermentation reduced the average particle size and increased the absolute value of zeta potential. It was visualized by SEM and CLSM maps that the macromolecular proteins in FSBM were broken down into fragmented pieces with a folded and porous surface structure. Fermentation increased the solubility, decreased the hydrophobicity, increased the free sulfhydryl content, decreased the antigenicity, improved the protein properties of SBM, and promoted further processing and production of FSBM as a food ingredient.

## 1. Introduction

SBM is the most important byproduct of soybean oil production from soybeans, with high protein content, is rich in nutrients, and contains a variety of essential amino acids [1], making it a high-quality plant protein source. However, SBM contains more structurally complex macromolecular proteins, which makes its protein digestibility and biological efficiency low [2]. Probiotic fermentation of soybean meal is an effective method for improving nutritional structure [3].

*Bacillus subtilis* strains are characterized by strong resistance, fast growth, relatively low nutritional requirements, and rapid secretion of large amounts of proteins and metabolites, and do not produce toxins, which makes them a safe fermentation microorganism [4]. *Bacillus subtilis* can degrade macromolecular proteins in SBM during its growth process, increasing protein solubility [5]. Differences in fermentation processes have a significant impact on the nutrient composition and final quality of FSBM [6], in which the change in acid-soluble protein content is one of the important indicators for the subsequent application of FSBM. Some of the soy proteins in soybean meal are inactive and need to be released via fermentation, whereby they are activated [7]. Increased acid-soluble protein content in FSBM is closely related to the desensitization of protein molecules, physiological activity, and digestive utilization [8]. Acid-soluble protein, which represents more active peptides produced by protein breakdown after fermentation, is often used to assess the degree of further processing of raw materials. The higher the content of acid-soluble protein, the better the digestion rate of the raw material. Hydrolysis destroys the protein structure of soybean meal, resulting in a significant decrease in its antigenicity [9].

Physical cracking such as the grinding of flour cannot release all of the nutrients in SBM. Even after cooking, a few nutrients remain inaccessible and unavailable to the human digestive system [10]. Fermentation improves soybean digestibility by physically and chemically breaking down indigestible substances locked into soybean seed structures and cells, or by enzymatically degrading indigestible polymers into monomers and their derivatives [11,12]. Dai et al. [13] used *Bacillus subtilis* fermented SBM and found that after fermentation, the secondary structure of the protein changed, making the protein molecules more loose and conducive to digestion and absorption. In addition, the various antinutritional factors contained in SBM limit its application in food products [14]. The antigenic proteins in SBM mainly include glycinin and β-conglycinin, whose special subunits make them stable and which cannot be removed by heating. Antigenic proteins have large molecular weights, which cannot be directly absorbed and utilized by the human body, reducing the efficiency of digestion, and even causing the body to develop allergies, causing abdominal pain and diarrhea or even death [15]. *Bacillus subtilis* can effectively decompose the antigenic and difficult-to-digest-and-utilize macromolecular proteins in SBM, improve the quality of SBM, and enhance the digestion and absorption rate [16]. Yin et al. [17] have shown that alkaline proteases produced by *Bacillus subtilis* exhibit relatively high efficiency in reducing the allergenicity of soybean proteins, making it possible to produce hypoallergenic soybean proteins. Therefore, fermentation of SBM using *Bacillus subtilis* is a research hotspot, and although there are some studies on its nutritional value, there are few related reports on the effect on the protein structure of SBM.

In conclusion, the aim of this study was to optimize the process parameters of solid-state fermentation of soybean meal by *Bacillus subtilis* to increase the acid-soluble protein content and improve the quality of SBM. Comparative analysis was conducted on the improvement of SBM protein structure and properties under optimal fermentation conditions, providing theoretical support for the actual production of FSBM.

## 2. Results

### 2.1. Optimization of the Fermentation Conditions

Table 1 illustrates the relationship between acid-soluble protein and the experimental factors using the quadratic regression equation:

Y = 12.78 + 0.76 × A−0.26 × B−0.31 × C−0.058 × A × B−0.39 × A × C + 0.003875 × B × C − 2.28 × A^2^ − 3.32 × B^2^ − 2.92 × C^2^

where Y is acid-soluble protein content (g/100 g), A is inoculation amount (%), B is time (h), and C is material-liquid ratio (g/mL).

As shown in Table 2, the established quadratic regression model is highly significant (*p* < 0.01), the misfit term is *p* = 0.1130, the misfit is not significant, and the model fits the results of the test values well. Meanwhile, the complex correlation coefficient of the regression equation, R^2^ = 0.9985, indicates that 99.85% variability can be explained by this response surface model.

The results of the analysis of variance (ANOVA) used to assess the effectiveness of the quadratic polynomial fit are presented in Table 2. The results show that the coefficient of determination (R^2^) of the quadratic polynomial was 0.9985, indicating that 99.85% of the total variation could be explained by the regression model. In this case, five secondary terms (A, AC, A2, B2, and C2) had a highly significant effect (*p* < 0.001) on the acidolysin content, as well as two metrics (B and C) (*p* < 0.01), and AC had a significant effect (*p* < 0.05).

**Figure 1:**
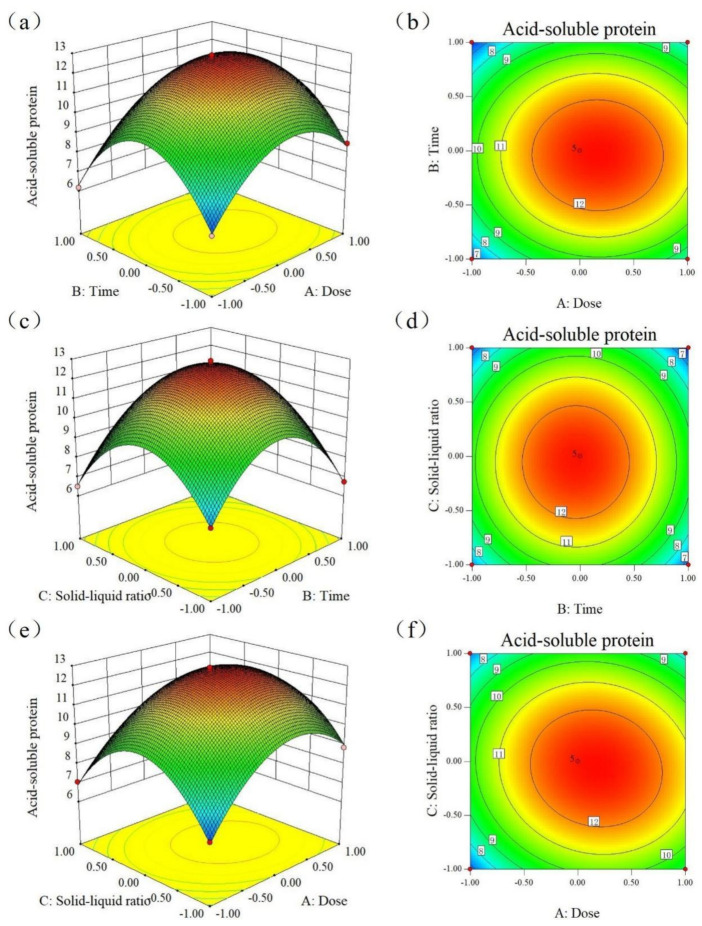
Response surface diagram and contour line diagram. Effect of inoculation amount and fermentation time on acid-soluble protein (**a**,**b**); material-liquid ratio and fermentation time on acid-soluble protein (**c**,**d**); inoculation amount and material-liquid ratio on acid-soluble protein (**e**,**f**).

In conjunction with Table 2, the shapes of the response surfaces and contour plots were observed to analyze the effects of the three factors on the acid-soluble protein content of SBM. Among them, see the significant degree of ellipse of contour plots in Figure 1 (*p* < 0.05), which indicates the significant effect of two cross-factors, the inoculum amount and the material-liquid ratio. The response surfaces were all convex with downward opening, the maximum value was predicted by the protrusion on the surface of the elliptical contour plots, and the optimal fermentation conditions were determined as: inoculum amount of 12.24%, time of 47.24 h, and material-liquid ratio of 1:0.586. Validated under these conditions, the acid-soluble protein content obtained was 12.88%, which was more in line with the model-predicted value of 12.86%, suggesting that the model could better reflect the actual situation of the optimization of the *Bacillus subtilis* fermentation process.

### 2.2. Protein Structure Analysis

#### 2.2.1. SDS-PAGE Protein Gel Electrophoresis

**Figure 2:**
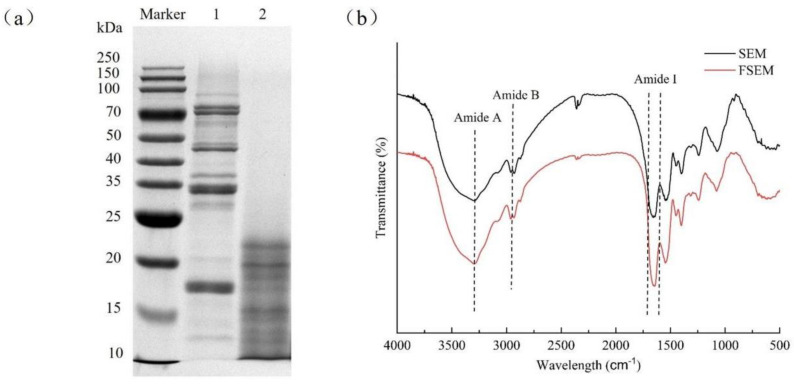
SDS-PAGE (**a**) and FT-IR (**b**). SBM for lane 1 and FSBM for lane 2.

The SDS-PAGE profiles of SBM before and after fermentation as well as the standard protein are shown in Figure 2a. After fermentation, the 25–250 KDa bands appeared to be significantly weakened, with multiple newborn peptide bands smaller than 25 KDa. Almost all of the large-molecule proteins were degraded into small-molecule products, which reduced the allergenic activity of SBM, and at the same time improved the digestibility of the proteins, because small-molecule proteins are more easily digested and absorbed by humans and animals.

#### 2.2.2. Fourier Transform Infrared Spectroscopy Assay

FTIR was used to analyze the changes in the chemical groups and secondary structure of the proteins, and the results of the infrared spectra of SBM and FSBM are shown in Figure 2b. The blue shift in the peak position of Amide A along with the decrease in the peak intensity of FSBM compared to SBM may be due to the fact that there is more O-H bond breakage during the fermentation process. Compared with SBM, the Amide B peak position of FSBM was red-shifted and the peak intensity was increased, which may be due to the C-H stretching vibration of CH3 and CH2 groups in the saturated chain structure of aliphatic amino acid side chains during the fermentation process, indicating that the fermentation may have increased the content of aliphatic amino acids.

The Amide I band is attributed to 80% C=O stretching vibration and 20% C-N stretching vibration, and the absorption peaks in this band mainly indicate inter- and intramolecular secondary structures of proteins. Compared with SBM, the Amide I peak position of FSBM was blue-shifted while the peak value was decreased, indicating that fermentation changed the protein spatial conformation, and the intramolecular robustness was broken. To further reveal the protein secondary conformation, we quantified the Amide I region using a deconvolution process, and the fitting analysis yielded Table 3. After fermentation, the content of α-helix was decreased by 8.3%, β-sheet was increased by 3.08%, β-turn was increased by 2.71%, and random coil was increased by 2.51% (*p* < 0.05). This indicated that fermentation changed the protein secondary structure from a rigid to flexible structure, improved the protein quality, and facilitated utilization by the organism.

#### 2.2.3. Particle Size Distribution

**Figure 3:**
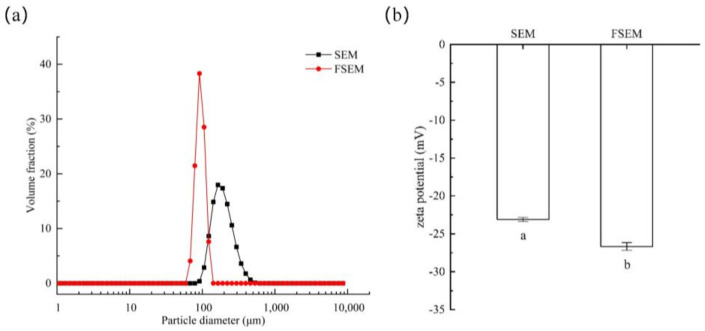
The particle size distribution of SBM and FSBM (**a**) and Zeta potential of SBM and FSBM (**b**). Different lowercase letters indicate significant difference at the level of *p* < 0.05 via SPSS 20.0 analysis.

The particle size distributions of SBM and FSBM are shown in Figure 3a, and the peak shapes are basically the same, but the peaks are different. The FSBM was overall shifted in the direction of smaller particle size. This may be due to the action of fermentation that destroys the ordered and compact structure of proteins in SBM, which reduces the average particle size and breaks it into smaller structures.

#### 2.2.4. Zeta Potential

Zeta potential is used to determine the strength of interparticle interaction forces. The dispersion or aggregation of protein molecules depends on the charge on their surface, which means that the breakdown of protein molecules increases the absolute value of the zeta potential [18]. As shown in Figure 3b, the significant increase in the absolute value of the zeta potential of FSBM indicates that the hydrolysis of proteins occurs during the fermentation process.

#### 2.2.5. Scanning Electron Microscopy (SEM)

**Figure 4:**
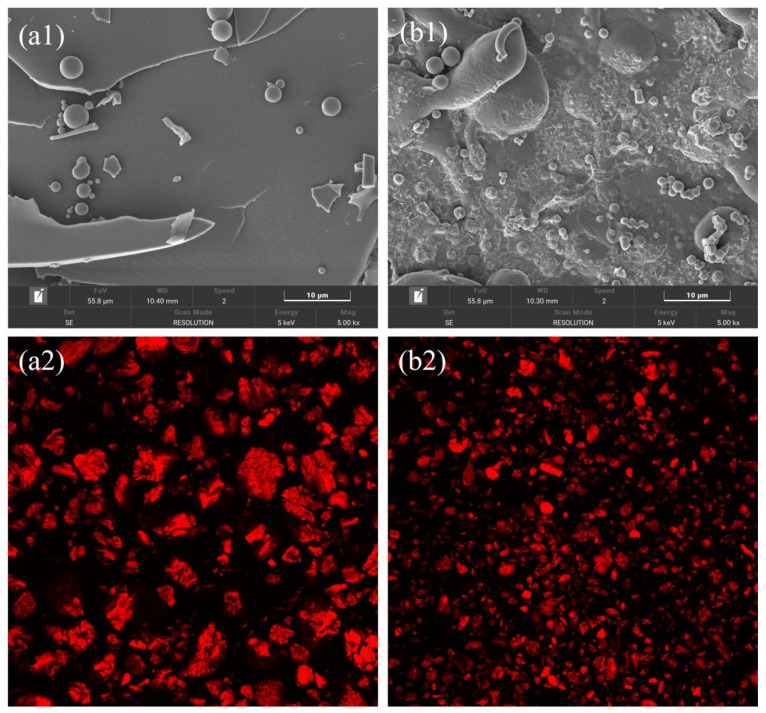
Surface morphology of SBM (**a1**) and FSBM (**b1**) using SEM (5000×). The surface morphology of SBM (**a2**) and FSBM (**b2**) was observed using CLSM.

From Figure 4(a1,b1), it can be seen that FSBM has a looser structure compared to SBM. At a magnification of 5000×, SBM presents as a larger lamellar structure with a flat surface, and the edges of the particles are smooth and basically void-free. In contrast, FSBM presents as a more fragmented and irregular lamellar structure, and the surface of the particles has been wrinkled and rendered porous under the action of *Bacillus subtilis*. The protein structure was more loose after fermentation, indicating that *Bacillus subtilis* was able to decompose the SBM material sufficiently, which made the protein more favorable for digestion and absorption, and also improved the nutritive property of SBM.

#### 2.2.6. Confocal Laser Scanning Microscopy (CLSM)

Figure 4(a2,b2) show the staining photos of SBM and FSBM samples recorded using CLSM. The protein particles of SBM were larger and had higher fluorescence intensity, while the protein particles of FSBM were fragmented into smaller particles with reduced fluorescence intensity. *Bacillus subtilis* probably firstly nibbled the protein on the surface of SBM to break up the bulky protein particles, which further increased the surface area of contact between the bacteria and proteins, degraded the macromolecular proteins from the outside to the inside, and further increased the digestibility and utilization of the raw materials.

### 2.3. Protein Properties Analysis

#### 2.3.1. Solubility of Protein and Surface Hydrophobicity

**Figure 5:**
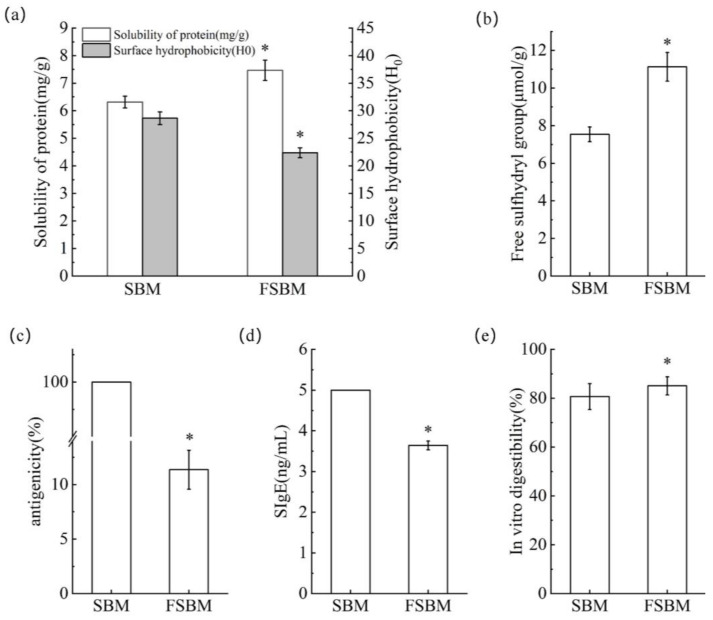
The degree of solubility (mg/g) and surface hydrophobic index (H0) of SBM and FSBM (**a**). Free sulfhydryl group (μmol/g) of SBM and FSBM (**b**). Protein antigenicity (%) of SBM and FSBM (**c**). Binding ability to human serum-specific antibodies (SIgE) (ng/mL) of SBM and FSBM (**d**). In vitro digestibilities (%) of SBM and FSBM (**e**). “*” indicates significant difference at the level of *p* < 0.05 via SPSS 20.0 analysis.

As shown in Figure 5a, the solubility of FSBM increases and conversely the surface hydrophobicity index decreases. This phenomenon indicates a negative correlation between the solubility of SBM and its surface hydrophobicity index, and this result is also consistent with the conclusion of Shon et al. [19]. The solubility of the protein is related to the hydrophobic structure of the protein surface, and the fermentation caused a change in the structure of SBM, which led to a decrease in the surface hydrophobicity index and an increase in solubility.

#### 2.3.2. Free Sulfhydryl Content

As shown in Figure 5b, the free sulfhydryl content in FSBM increased by 47.67% compared to SBM. This may be due to the effect of fermentation, which denatured the protein, increased disulfide bond breakage, and free sulfhydryl groups to the surface.

#### 2.3.3. Antigenicity of Proteins

The antigenicity of SBM before and after fermentation by *Bacillus subtilis* is shown in Figure 5c. As can be seen from the figure, the protein antigenicity was significantly reduced (90.1%) after fermentation by *Bacillus subtilis* natto. Fermentation caused large changes in the antigenic epitopes of the proteins. These epitopes may be destroyed by degradation during fermentation, or conformational changes may occur to bury them.

#### 2.3.4. Binding Ability to Human Serum-Specific Antibodies (SIgE)

The binding capacity of FSBM protein of *Bacillus subtilis* natto under optimal fermentation conditions, with human serum-specific antibody, SIgE, is shown in Figure 5d. The binding capacity of FSBM with serum-specific SIgE after fermentation showed a significant decrease compared to that before fermentation (*p* < 0.05).

The content of serum-specific antibodies in humans reacts to the level of immunoglobulins, which have antiviral and anti-infectious functions. Fermentation breaks down the proteins of SBM into peptides and amino acids, which reduces the binding sites of specific antibodies, destroys their antigenic epitopes, and reduces allergic reactions.

#### 2.3.5. In Vitro Digestibility

The in vitro digestibility of SBM and FSBM is shown in Figure 5e. The in vitro digestibility of FSBM was significantly higher than that of SBM. This may be attributed to the high content of acid-soluble proteins in FSBM after the optimization of the fermentation conditions, which indicates the presence of a higher amount of small peptides and amino acids, which, in turn, improves the in vitro digestibility of the protein components.

## 3. Discussion

The content of acid-soluble protein is an important indicator for evaluating the nutritional quality of fermented soybean meal. From Table 2, it can be seen that the primary terms A,B,C and secondary terms A2,B2,C2 have highly significant effects on the acid-soluble protein content, the interaction term AC has a significant effect on the acid-soluble protein content, and the other factors are not significant. Comparing the magnitude of F, it is easy to obtain the order of factors affecting the acid-soluble protein content as follows: Inoculation amount > Material-liquid Ratio > Fermentation time. The optimal process parameters obtained in this study were compared with the fermentation time (62.32 h) of Zhang et al. [20]. The process obtained in this study had a shorter fermentation time, which saved energy and improved efficiency.

From the structural study of SBM using SDS-PAGE, FTIR, and particle size analysis, it can be concluded that most of the large-molecule proteins were degraded into small-molecule proteins after fermentation. The SDS-PAGE pattern showed that the 25~250 KDa bands were basically invisible, and it was reported that *B. nattoensis* could reduce the large-molecule proteins, such as 7s, 11s, etc., in soybean by 80%, according to Zheng et al. [5]. FTIR was used to analyze the changes of chemical groups and secondary structure of proteins, in which the Amide A peak correlates to adsorbed water O-H stretching vibration and N-H stretching vibration, reflecting the level of C=O...H-N hydrogen bonding within the protein molecule and H-O...N-H hydrogen bonding between molecules, and the strength of the hydrogen bonds reflects the O-H stretching vibration of water molecules or the amount of adsorbed water [21]. The blue shift of the Amide A peak of FSBM may be related to the need to utilize water for the vital activities of the bacteria during the fermentation process. The absorption peaks of the Amide I band mainly indicate the secondary structure of the protein. α-helix and β-sheet have a significant rigidity in the lamellar structure with more hydrogen bonds present. The β-turn and random coil, on the other hand, exhibit great flexibility because of the greater degrees of freedom between peptides. α-helix content and α-helix/β-sheet were reduced in FSBM. Microbial fermentation to hydrolyze the α-helix component of proteins is important because it indicates that the digestibility of fermented soybean meal is likely to be improved [22]. Wang et al. [23] demonstrated that a reduction in the α-helix component was positively correlated with increased protein digestibility. Alrosan et al. [24] found a negative correlation between α-helix/β-sheet ratio and protein digestibility.

In FSBM, the content of another rigid structure, the β-sheet structure, increased significantly, and the change in the content of the β-sheet structure was correlated with functional activity. The fermentation process may cause changes in the β-sheet structure of proteins, which is favorable for the stability of the expression of their physiological activity [25,26]. β-turn and random coil content were both significantly increased in FSBM, and these changes were positively correlated with the improvement in protein quality [27]. Similar results were obtained by Zheng et al. [5]: FSBM significantly reduced ANF (anti-nutritive factor) content and improved its protein digestibility with an increase in the percentage of RC and a decrease in the percentage of α-helix, which was determined by FTIR spectroscopy. The particle size of FSBM was significantly reduced and the zeta potential was significantly increased, indicating that FSBM was more thoroughly hydrolyzed and formed smaller molecular protein structures. This reduction in particle size may be attributed to the disruption of insoluble protein aggregates via fermentation, leading to the exposure of polar amino acids, which in turn increases the protein surface charge, intermolecular electrostatic repulsion, and inhibition of protein aggregation [28]. Combined with SEM micrographs and CLSM maps, it was visually observed that the surface of SBM was eroded by the bacteria, with rough surface and smaller particles, which might be related to the protein-degrading enzymes in the fermentation products [8]. Similar results were obtained by Dai et al. [13]: the fermentation of SBM by *Bacillus subtilis* decreased the α-helix content and increased the random coil content, and the fermentation could obtain a loose protein structure with a significantly reduced particle size and uniform dispersion of protein particles as measured by atomic force microscopy (AFM). Proteins with smaller particles have higher solubility, which is consistent with the results of this experiment and may be more favorable for human and animal absorption [29].

Compared with SBM, the protein properties of FSBM were significantly improved. Sulfhydryl groups are important functional groups in soybean proteins, and the content of sulfhydryl groups is related to protein denaturation, and other functional properties change accordingly. The free sulfhydryl group content of FSBM was significantly increased, indicating that the fermentation treatment can disrupt the internal structure of soybean protein molecules, resulting in the unfolding of part of the structure, which in turn exposes sulfhydryl groups hidden inside the protein molecules to the surface of the molecules [30]. The FSBM solubility increased and surface hydrophobicity decreased. Huang et al. [31] showed that protein solubility was positively correlated with digestibility and surface hydrophobicity was negatively correlated with digestibility. We also obtained similar results that the in vitro digestibility of FSBM was significantly higher than that of SBM. Due to the good degradation of SBM by *Bacillus subtilis*, its antigenic protein structure was sufficiently disrupted. Song et al. [32] found that Lactobacillus plantarum fermentation of soybean meal resulted in an 88–89% reduction in the antigenicity of soybean proteins. The antigenicity of SBM was also reduced by 88–89% by *Bacillus subtilis* which reduced the antigenicity of SBM more favorably than Lactobacillus plantarum. Yang et al. [33] verified via in vitro competitive inhibition ELISA and mouse models that FSBM causes less intestinal damage, which may be related to the hydrolysis of soy protein allergen sequences N232-D383, G253-I265, E169-S215, G68-G98, A365-I375, and V153-A167. In conclusion, the protein structure of FSBM is looser than that of SBM, the surface is rougher with more voids, the antigenicity is reduced, and the digestibility is elevated, which is more conducive to the production and processing of SBM, and the utilization value of SBM is improved.

## 4. Materials and Methods

### 4.1. Materials

Soybean meal (55 °C, 24 h drying, and standby) was provided by Jilin Chucai Agricultural Products Development CO. LTD in Huadian, Jilin Province, China. The *Bacillus subtilis* (CGMCC 11164) strain was isolated and screened from homemade pickled cabbage juice by the food microbiology team of Agricultural Products Processing Institute of Jilin Academy of Agricultural Sciences, and was stored in our laboratory. All other chemicals were analytically pure or had higher purity.

### 4.2. Preparation of the Culture Medium

LB liquid culture medium (g/L): 10.0 g of peptone, 3.0 g of yeast extract, 3.0 g of glucose, and 5.0 g of NaCl, pH 6.2–6.4.

### 4.3. Preparation of Fermented Soybean Meal

#### 4.3.1. Strain Culture Method

Seed solution preparation: 200 mL of LB liquid culture medium in a 500 mL tapered flask, at 121 °C, with 21 min of high-temperature autoclaving. Take a single colony and inoculate it into the medium at 37 °C with 195 rpm/min oscillation of the culture for 12 h, and make a seed liquid. Transfer it to the inoculum of 5% LB medium, at 37 °C with 195 rpm/min of shaking of the culture for 4 h, and expand the culture.

#### 4.3.2. RSM

RSM was used to explore the relationship between the three controlled experimental variables (including fermentation inoculation amount, time, material liquid ratio) and the response value (acid-soluble protein content). After obtaining the appropriate range of these independent variables using univariate experiments, a RSM with three factors and three levels was generated by the Box-Behnken design (BBD).

Soybean meal that had been milled and sieved through a 40-mesh sieve was inoculated with starter culture (>7 log CFU per mL of cells) in a container covered with two layers of moist gauze at 37 °C. Other conditions were set according to the optimized conditions above. After fermentation, the FSBM samples were dried in a dry and ventilated place for 24 h and stored at −20 °C [5]

Response surface methodology (RSM) combined with a one-factor test was used to optimize the fermentation conditions using acid-soluble protein content as the response value. The specific factors and levels are shown in Table 4.

### 4.4. Structural Analysis

#### 4.4.1. Protein Preparation

The sample powder of SBM and FSBM was dissolved in distilled water at a ratio of 1:10 *w*/*w*. The pH was adjusted to 8.0 and the solution was stirred with a magnetic stirrer for 2 h. The powder solution was centrifuged at 10,000× *g* for 30 min and the supernatant was collected. The pH of the supernatant was adjusted to 4.5 and the precipitate was obtained via centrifugation at 10,000× *g* for 30 min. The precipitate was redissolved in distilled water with the pH adjusted to 7.0. The solution was finally lyophilized using a freeze dryer and stored at 4 °C as a backup.

#### 4.4.2. Sodium Dodecyl Sulfate-Polyacrylamide Gel Electrophoresis (SDS-PAGE)

The samples were dispersed at 1 mg/mL in 5 × loading buffer, denatured at 100 °C for 10 min, and centrifuged at 10,000 rpm for 10 min, and 10 μL of supernatant was added to the comb wells. Electrophoresis was carried out in a concentration gel (4%) at 80 V/gel for 20 min and in a separation gel (12%) at 120 V/gel for 60 min. The bands were visualized after staining and washing with Caumas Brilliant Blue R-250 [34].

#### 4.4.3. FT-IR

Refer to the method of Yasar et al. [22]. The SDF chemical composition changes were analyzed using an FT-IR spectrometer (Nicolet 6700 of Thermo Fisher Scientific, Waltham, MA, USA). Soybean lag samples were mixed with KBr (1:100 *w*/*w*) for grinding and pressing. The mixture was immediately placed in the optical path for scanning, and 400–4000 cm^−1^ FT-IR spectra were recorded over a range via 32 scans with a resolution of 4 cm^−1^.

#### 4.4.4. Determination of Particle Size and Zeta Potential

Refer to the method of Liu et al. [35]. Protein samples were dispersed in sterile water at (1 mg/mL) and analyzed using a Litesizer™ 500 particle size analyzer (AntonPaar, Graz, Austria) with parameter settings according to Lu et al. [18].

#### 4.4.5. SEM Analysis

The microstructure of the soybean residue sample was observed using SEM (Zeiss SUPRA5, Oberkochen, Germany). Before testing and recording, soybean residue samples were attached to metal plates using conductive tape to facilitate sputtering gold plating. Each micrograph was taken at a 1000× and 3000× magnification at 10 kV.

#### 4.4.6. CLSM Analysis

Samples for CLSM analysis were stained with fluorescent dye rhodamine B [0.001% (*w*/*v*)] for 20 min [36]. Then, the stained sections were observed using an Olympus Fluoview FV30000 Laser Scanning Confocal Microscope (Olympus Corp., Tokyo, Japan). The excitation wavelength of the laser was 561 nm. Using the staining results, the specific location and detailed distribution status of the protein components could be obtained [37].

### 4.5. Protein Properties Analysis

#### 4.5.1. Solubility of Protein

The protein solubility was determined by the method of Samoto et al. [38]. The protein samples were dissolved in 10 mL of distilled water, stirred magnetically for 30 min, and centrifuged at 10,000 r/min for 20 min at 4 °C. The supernatant was extracted, and the protein content was determined using the Lowry method after moderate dilution. The solubility of protein was expressed as the mass concentration (mg/g) of protein content in the supernatant to the total protein content.

#### 4.5.2. Surface Hydrophobicity

The surface hydrophobicity of fermented soybean meal proteins was determined using the fluorescent probe 8-anilino-1-cairosulfonic acid (ANS) method. Protein solutions of soybean meal at pH 7 and 1.0% were prepared with phosphate buffer at pH = 7 to 0.004%, 0.008%, 0.012%, 0.016%, and 0.02%, respectively, with 20 μL of 8 mM ANS added to 4 mL of the protein solutions in centrifuge tubes and mixed well. The fluorescence intensity was measured using a fluorescence spectrophotometer at an excitation wavelength of 370 nm and emission wavelength of 490 nm, respectively, and the concentration of protein was used as the horizontal coordinate and the fluorescence intensity as the vertical coordinate, and the initial slope was the surface hydrophobicity index.

#### 4.5.3. Free Sulfhydryl Group Content

Refer to the method of Beveridge et al. [39] for determination. Sulfhydryl content was measured using a micro sulfhydryl kit.

#### 4.5.4. Protein Antigenicity

The changes in immunoreactivity of soy protein before and after fermentation were determined using the RIDASCREENR FAST Soya kit. The protein concentration of the samples was adjusted to 2.5~20 mg/kg according to the sandwich enzyme-linked immunosorbent assay (sandwich ELISA). Absorbance values were measured at 450 nm. Using the protein concentration as the horizontal coordinate and the absorbance value as the vertical coordinate, a standard curve was plotted according to the standard, and the antigenicity of the sample was calculated.

#### 4.5.5. Determination of Binding Ability to Human Serum-Specific Antibodies (SIgE)

Detection was performed using the human serum-specific (SIgE) kit. The ability of SBM and FSBM to bind to specific SIgE in human serum was assayed according to the ELISA method, with appropriate modifications following the experimental method of Peñas et al. [40].

#### 4.5.6. In Vitro Digestibility

In vitro digestion assay using extracted proteins. The in vitro digestibility of proteins and the rate of nitrogen release during digestion were determined by the method of Tang [41].

### 4.6. Statistical Analysis

SPSS 20.0 (IBM Corporation, Armonk, NY, USA) was used to process the data. The response surface design was performed using Design Expert 11.0.4. Chart mapping was completed using Origin 9.0. The results of all graphical experiments are expressed as mean ± standard deviation, with significant differences at *p* < 0.05 and highly significant differences at *p* < 0.01, which was considered statistically significant [42].

## 5. Conclusions

The optimal fermentation conditions for the solid-state fermentation of soybean meal by *Bacillus subtilis* obtained in this study are favorable for saving production energy and controlling costs. Compared with SBM, the protein secondary structure of FSBM was more flexible, and the rigid structure was transformed into a flexible structure. From the SEM and CLSM images, it was shown that FSBM had more folds and void structures on the surface, and the surface protein particles were degraded with weaker fluorescence intensity. In terms of protein properties, FSBM showed increased solubility, weakened hydrophobicity, and reduced antigenicity, which is more favorable for digestion and absorption. Combining the changes in protein structure and properties, we can conclude that the nutritional quality of SBM was optimized and the nutritional value was improved after solid-state fermentation using *Bacillus subtilis*. It lays the foundation for the re-exploration of FSBM as a functional food ingredient.

## Figures and Tables

**Table 1:** Experimental data of the three-factor, three-level Box-Behnken design for RSM and study response.

Runs	Inoculation Amount (%)	Time (h)	Material-Liquid Ratio (g/mL)	Acid-Soluble Protein Content (g/100 g)
1	−1.00	0.00	1.00	7.0518
2	0.00	1.00	−1.00	6.7463
3	−1.00	−1.00	0.00	6.6209
4	0.00	1.00	1.00	6.0381
5	0.00	0.00	0.00	12.7656
6	−1.00	1.00	0.00	6.1621
7	0.00	0.00	0.00	12.7742
8	1.00	−1.00	0.00	8.4825
9	0.00	−1.00	1.00	6.4956
10	0.00	−1.00	−1.00	7.2193
11	0.00	0.00	0.00	12.9152
12	1.00	1.00	0.00	7.7903
13	−1.00	0.00	−1.00	6.8049
14	1.00	0.00	1.00	7.5611
15	1.00	0.00	−1.00	8.8643
16	0.00	0.00	0.00	12.6207
17	0.00	0.00	0.00	12.8134

**Table 2:** ANOVA with the response face quadratic model.

Source	Sum of Squares	Df	Mean Square	F Value	*p* Value	Significance ^1^
Model	120.04	9	13.34	534.27	<0.0001	***
A-Inoculation amount	4.59	1	4.59	183.79	<0.0001	***
B-Time	0.54	1	0.54	21.69	0.0023	**
C-Material-liquid Ratio	0.77	1	0.77	31.00	0.0008	**
AB	0.014	1	0.014	0.55	0.4842	
AC	0.60	1	0.60	24.06	0.0017	*
BC	6.006 × 10^−5^	1	6.006 × 10^−5^	2.406 × 10^−3^	0.9622	
A^2^	21.97	1	21.97	879.90	<0.0001	***
B^2^	43.92	1	43.92	1759.36	<0.0001	***
C^2^	35.98	1	35.98	1441.21	<0.0001	***
Residual	0.17	7	0.025			
Lack of Fit	0.13	3	0.043	3.85	0.1130	
Pure Error	0.045	4	0.011			
Cor total	120.22	16	R^2^ = 0.9985			

^1^ Significance: * *p* < 0.05; ** *p* < 0.01; *** *p* < 0.001.

**Table 3:** Secondary structure relative content of original SBM and FSBM protein samples.

Sample	α-Helix (%)	β-Sheet (g/100 g)	β-Turn (%)	Random Coil (%)	Ratio ^1^ (%)
SBM	24.45 ± 0.18 a	29.51 ± 0.67 a	33.16 ± 0.10 a	12.88 ± 0.79 a	82.85
FSBM	16.15 ± 0.07 b	32.59 ± 0.76 b	35.87 ± 0.93 b	15.39 ± 0.61 b	48.55

^1^ Ratio of α-helix: β-sheet. Data are expressed as means ± standard deviation of three independent replicates. Different lowercase letters indicate significant difference at the level of *p* < 0.05 via SPSS 20.0 analysis.

**Table 4:** Factors and levels in the response surface analysis.

Levels	Inoculation Amount (%)	Time (h)	Material-Liquid Ratio (g/mL)
−1	9	24	1:0.4
0	12	48	1:0.6
1	15	72	1:0.8

## Data Availability

Data are contained within the article.
